# Recombinant Thermostable DNA Polymerases: Current Approaches to Production, Molecular Engineering, and Applications in Biotechnology and Diagnostics

**DOI:** 10.3390/ijms27167188

**Published:** 2026-08-11

**Authors:** Arman Mussakhmetov, Bekbolat Khassenov

**Affiliations:** National Center for Biotechnology LLP., building 13/5, Korgalzhyn Highway, 010000 Astana, Kazakhstan; mussakhmetov@biocenter.kz

**Keywords:** DNA polymerases, thermostable polymerases, recombinant proteins, protein engineering, Taq, Pfu, Hot Start, PCR, LAMP, molecular diagnostics

## Abstract

DNA polymerases are crucial for the replication and repair of genetic material. Advances in recombinant DNA technology and protein engineering have enabled the production of enzymes with specific catalytic properties tailored to the requirements of molecular diagnostics, next-generation sequencing, and synthetic biology. This review discusses the classification and structural–functional organization of DNA polymerases, with an emphasis on the thermostable members of Families A and B, which are of great practical importance. The main systems for heterologous expression and methods for purifying recombinant polymerases are summarized. Molecular engineering approaches, including rational design, site-directed mutagenesis, directed evolution, and domain engineering, are also discussed, highlighting how enzymes with improved synthesis fidelity, processivity, inhibitor resistance, and broadened substrate specificity are created. Technologies for developing hot-start polymerases along with the creation of chimeric and multifunctional polymerases are reviewed. Information on commercial polymerases utilized in scientific research and molecular diagnostics is also provided. Furthermore, the current applications of recombinant DNA polymerases in conventional, quantitative, and digital PCR; isothermal amplification; sequencing; synthetic biology; and molecular diagnosis of infectious and hereditary diseases are summarized. Finally, we discuss how the integration of structural biology, computational modeling, and high-throughput screening creates new prospects for engineering next-generation specialized enzymes.

## 1. Introduction

DNA polymerases are key enzymes involved in the replication and repair of genetic material and facilitate the synthesis of complementary strands based on DNA template sequences. Owing to their ability to catalyze the specific synthesis of nucleic acids, these enzymes have become an integral part of modern molecular biology, biotechnology, and medical and veterinary diagnostics. DNA polymerases are widely used for cloning, sequencing, site-directed mutagenesis, DNA barcoding, nucleic acid labeling, amplification of genetic material, and other molecular biological operations [1,2,3,4].

DNA polymerases are a special class of molecular machinery that transfer genetic information between generations of cells in accordance with the Watson–Crick rule of base complementarity [5]. The processes of template recognition, nucleotide selection, and phosphodiester bond formation are controlled by a series of structural and kinetic checkpoints that ensure highly accurate DNA synthesis [6,7], and the “steric gate” mechanism ensures substrate selectivity, preventing the incorporation of ribonucleotides into the growing DNA strand [8,9].

The history of DNA polymerase research dates to the discovery of DNA polymerase I in *Escherichia coli* cells by Kornberg et al., who first demonstrated template-directed DNA synthesis in vitro [10,11]. These studies have paved the way for the development of cutting-edge technologies for nucleic acid sequencing and amplification.

Despite their common functions in DNA synthesis, different polymerases vary in their catalytic properties, including thermal stability, half-life, replication accuracy, processivity, and substrate specificity. Some polymerases (Pfu, Vent) possess 3′→5′ exonuclease activity, which enables the correction of replication errors, whereas others (Taq, Tth) lack this mechanism, leading to a higher frequency of nucleotide mismatches [12].

The discovery of thermostable DNA polymerases is of particular importance in molecular biology. For example, the introduction of Taq polymerase from *Thermus aquaticus* has made it possible to automate the polymerase chain reaction (PCR), making it one of the most widely used methods for analyzing nucleic acids [13,14]. Subsequently, numerous polymerases from other thermophilic bacteria and archaea were identified, including Tth, Pfu, Vent, Deep Vent, KOD, and Bst, which have greatly expanded the possibilities for DNA amplification due to their combination of high thermal stability, processivity, and, in some cases, proofreading activity [1,15].

The demand for DNA polymerases has increased recently. Modern PCR methods, including quantitative PCR (qPCR), digital PCR (dPCR), capillary sequencing, next-generation sequencing (NGS), and isothermal amplification, often involve very small amounts of templates and complex clinical and environmental samples containing various inhibitors that require a combination of high accuracy, fast synthesis rates, and high processivity [16,17,18]. Natural DNA polymerases do not fully meet these requirements. Consequently, molecular engineering approaches focused on developing recombinant polymerases with improved characteristics are being actively investigated.

Current modification strategies include rational design, site-directed mutagenesis, directed evolution, the generation of hybrid enzymes, and combined polymerase systems. These findings have led to the development of a new generation of highly precise enzymes that are resistant to inhibitors, capable of efficiently amplifying long GC-rich DNA regions, and functioning within modern diagnostic platforms [1,19].

The objective of this review is to analyze the current approaches for the production and engineering of recombinant DNA polymerases used in molecular biology, biotechnology, and diagnostics. This review focuses on expression systems, strategies for the molecular modification of enzymes, and their use in various PCR applications, sequencing, and other modern molecular diagnostic technologies.

## 2. Polymerase Classification

DNA polymerases represent an extensive and evolutionarily ancient group of enzymes that are classified based on amino acid sequence homology and structural similarity of their catalytic subunits. Seven major families of DNA polymerases have been identified: A, B, C, D, X, Y, and RT (Table 1). This classification reflects not only enzyme origin but also functional specialization. DNA polymerase families differ in their roles, including chromosomal DNA replication, DNA damage repair, translesion synthesis, Okazaki fragment processing, and RNA-templated DNA synthesis [8,20,21,22,23,24].

Family A polymerases include bacterial Pol I-like enzymes involved in DNA repair, removal of RNA primers, and Okazaki fragment processing, bacteriophage polymerases, and eukaryotic mitochondrial Pol γ. The prototype of this family is *Escherichia coli* DNA polymerase I, and it has been a crucial part of numerous advances in molecular biology [10]. Notable thermostable members of Family A include the Taq, Tth, and Bst polymerases from the thermophilic bacteria *Thermus aquaticus* [13], *Thermus thermophilus* [59], *Geobacillus stearothermophilus* [60], respectively. Taq polymerase has become the basis of classical PCR, whereas Bst polymerase, owing to its strand-displacement ability, is widely used in isothermal amplification methods such as loop-mediated isothermal amplification (LAMP) [61]. Despite the lack of proofreading 3′→5′ exonuclease activity in most commercially available Family A enzymes, their high synthesis rate, thermal stability under repeated cycling, and technological convenience have made them indispensable in routine PCR diagnostics [62,63,64,65].

In addition to canonical replicative enzymes, metazoan genomes encode specialized A-family polymerases that lack proofreading ability and function in specific DNA repair and damage-tolerance pathways. These polymerases include Pol θ (POLQ) and Pol ν (POLN). Pol θ has a unique dual-domain structure that combines a N-terminal helicase-like/ATPase region with a C-terminal polymerase domain. Its low fidelity and ability to bypass various lesions, such as abasic sites and thymine glycol, are key to its primary cellular roles in microhomology-dependent theta-mediated end joining (TMEJ) and translesion synthesis (TLS) [66,67]. In contrast, Pol ν exhibits strong strand-displacement activity and restricted metazoan distribution. Although it can bypass select major-groove adducts in vitro, genetic evidence from in vivo studies indicates that Pol ν does not function as a general crosslink-repair polymerase. Rather, it plays a specialized role in processing complex looped-out or hairpin structural intermediates during meiotic homologous recombination [68].

Family B includes major eukaryotic replicative polymerases, most archaeal enzymes, and several viral replicases. Unlike most enzymes in Family A, members of Family B typically possess 3′→5′ exonuclease activity, which enables proofreading and, consequently, higher accuracy in DNA synthesis. This family includes Pfu, Vent, Deep Vent, KOD, Tgo, 9°N, and Pab DNA polymerases from *Pyrococcus furiosus* [69], *Thermococcus litoralis* [70,71], *Pyrococcus* sp. GB-D [72], *Pyrococcus* sp. KOD1 [73], *Thermococcus gorgonarius* [74], *Thermococcus* sp. 9°N-7 [72], and *Pyrococcus abyssi* [75], respectively, which are widely used for high-precision amplification, cloning, site-directed mutagenesis, and library preparation for NGS. At the evolutionary level, the PolB catalytic core belongs to the RRM/palm lineage and shows affinity with reverse transcriptases and viral RNA-dependent RNA polymerases, indicating deep evolutionary links between different classes of template-dependent polymerases [21,22,76,77,78,79].

Stress-responsive polymerases within the B-family display contrasting fidelity profiles and mechanistic roles in damage tolerance across prokaryotes and eukaryotes. Bacterial Pol II (polB), an SOS-inducible single-subunit enzyme, has proofreading 3′→5′ exonuclease activity and serves as a high-fidelity, predominantly antimutagenic backup to Pol III during replication restart and lesion clearance [80,81]. In contrast, eukaryotic Pol ζ is a four-subunit complex that lacks proofreading ability. It comprises the catalytic Rev3/REV3L and Rev7/MAD2L2 subunits, as well as the POLD2–POLD3 accessory subunits. Pol ζ primarily functions as a specialized “extender” polymerase during translesion synthesis (TLS). It elongates from mismatched or lesion-opposite termini. Often operating in concert with Rev1 and inserter polymerases, Pol ζ drives much of damage-induced mutagenesis while performing essential developmental functions. This is evidenced by embryonic lethality in REV3-knockout mice [66,82].

**Figure 1:**
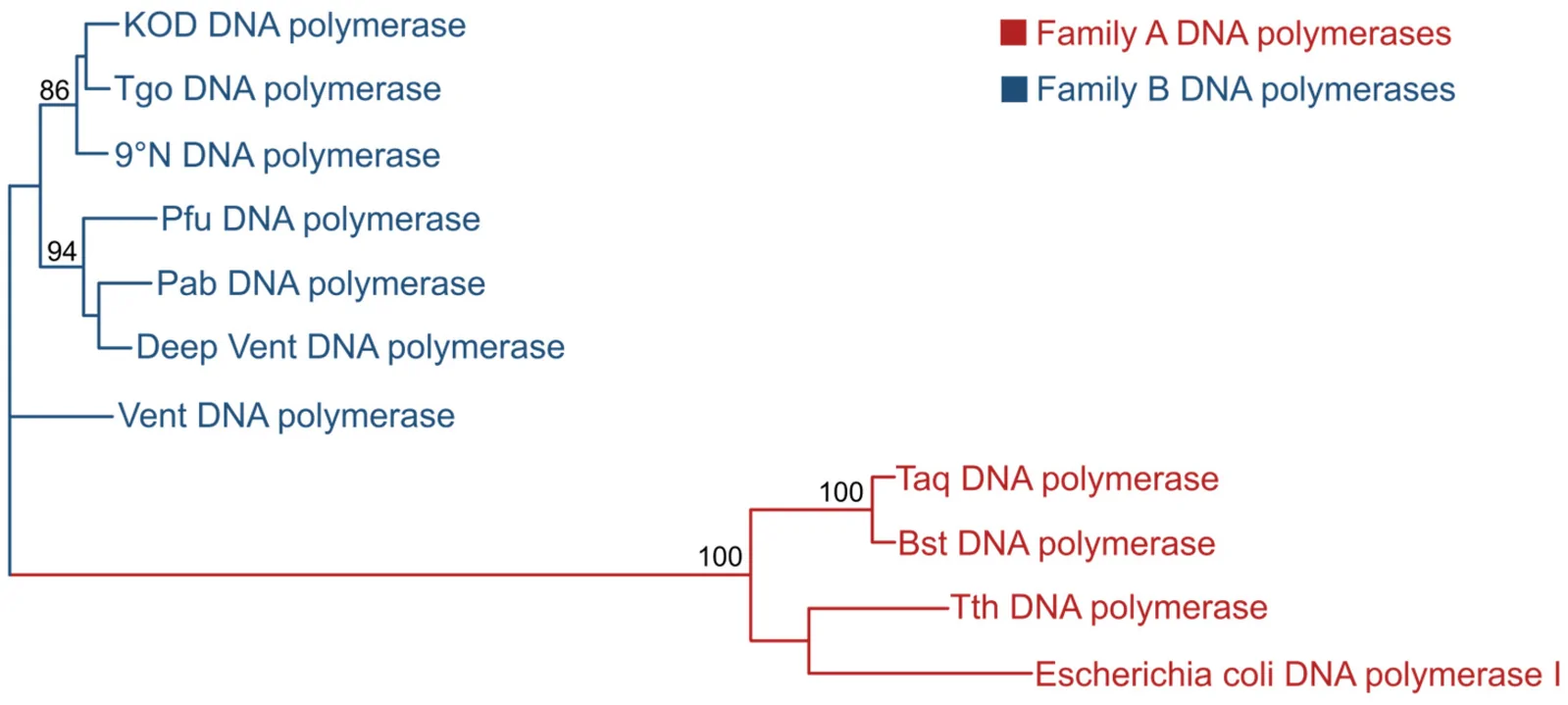
Phylogenetic tree of representative thermostable DNA polymerases from families A and B reconstructed using the Maximum Likelihood method based on amino acid sequences. Numbers at the nodes indicate bootstrap support values (%) obtained from 1000 bootstrap replicates. Branch lengths are shown schematically and are not proportional to evolutionary distances. Protein sequence identifiers used for phylogenetic reconstruction were as follows: Taq DNA polymerase (*Thermus aquaticus*, GenBank: AAA27507.1), Tth DNA polymerase (*Thermus thermophilus*, GenBank: BAA06033.1), Bst DNA polymerase (*Geobacillus stearothermophilus*, GenBank: AAB62092.1), *Escherichia coli* DNA polymerase I (GenBank: ELL39752.1), Pfu DNA polymerase (*Pyrococcus furiosus*, GenBank: BAA02362.1), Pab DNA polymerase (*Pyrococcus abyssi*, PDB: 4FLU chain A), KOD DNA polymerase (*Thermococcus kodakarensis*, PDB: 1WN7 chain A), Vent DNA polymerase (*Thermococcus litoralis*, GenBank: ADK47977.1), Deep Vent DNA polymerase (*Pyrococcus* sp. GB-D, PDB: 5H12 chain A), 9°N DNA polymerase (*Thermococcus* sp. 9°N-7, GenBank: AAA88769.1), and Tgo DNA polymerase (*Thermococcus gorgonarius*, RefSeq: WP_088885078.1).

Phylogenetic analysis of thermostable polymerase sequences from families A and B revealed that they were divided into two major clusters (Figure 1). Family A polymerases, represented by Taq, Tth, Bst, and Eco Pol I, form a distinct bacterial branch, whereas archaeal polymerases Pfu, Pab, Deep Vent, Vent, KOD, Tgo, and 9°N are grouped in the Family B cluster. Within Family B, additional subgroups that reflect the phylogenetic proximity of the host organisms can be identified. For example, KOD, Tgo, and 9°N are grouped with members of the genus *Thermococcus*, whereas Pfu, Pab, and Deep Vent form a closely related group of polymerases associated with the genus *Pyrococcus*. This classification is consistent with the differences in catalytic properties, proofreading activities, and practical applications.

Polymerases of Family C are represented by the major bacterial replicases, DnaE and PolC, which are components of DNA polymerase III. These enzymes play a key role in bacterial genome replication; however, they currently have limited practical significance compared to enzymes from families A and B [20,41,83]. Family D includes the archaeal replicative polymerases PolD, consisting of a catalytic subunit (DP2) and a small subunit (DP1) with 3′→5′ exonuclease activity. The unique DPBB architecture of PolD brings these enzymes closer to multisubunit RNA polymerases and makes them important subjects for studying the early evolution of replication systems [79,84,85,86].

The X and Y families are primarily involved in DNA repair. Polymerases of the Family X, including Polβ, Polλ, Polμ, and TdT, participate in base excision repair (BER), non-homologous end joining, and V(D)J recombination [47,49,87,88]. In contrast, the Family Y polymerases enable translesion synthesis through damaged DNA regions. Their open and flexible active site allows them to bypass damage in the DNA template; however, this is accompanied by a high error rate and a lack of 3′→5′ proofreading activity [21,89,90,91].

Reverse transcriptases, which belong to the RT family, constitute a distinct group. These enzymes catalyze DNA synthesis using an RNA template and play vital roles in the life cycles of retroviruses, retrotransposons, and telomerases. In biotechnology, reverse transcriptases form the basis for RT-PCR, RNA-seq, gene expression analysis, and diagnosis of RNA-containing viruses. Thermostable variants of RT are of particular interest, including mutant forms of MMLV RT, HIV-1 RT, and bacterial TGIRT enzymes, which can function effectively at elevated temperatures and replicate structured RNA templates [92,93,94,95,96,97].

Thus, the classification of DNA polymerases reflects their evolutionary origins, structural organizations, and functional specializations. However, from an application perspective, the thermostable polymerases of families A and B are of great importance. Enzymes of Family A, primarily Taq, Tth, and Bst, have driven the development of routine PCR, qPCR, and isothermal amplification, whereas archaeal polymerases of Family B, including [69,70,72,73,74], have become the foundation of high-precision amplification, cloning, sequencing, and molecular engineering. The combination of thermal stability, processivity, proofreading activity, and the ability to undergo targeted modification makes these two families central to the development of modern recombinant enzymes for molecular diagnostics, genomic research, and biotechnology.

## 3. Structural and Functional Organization of Thermostable DNA Polymerases

**Figure 2:**
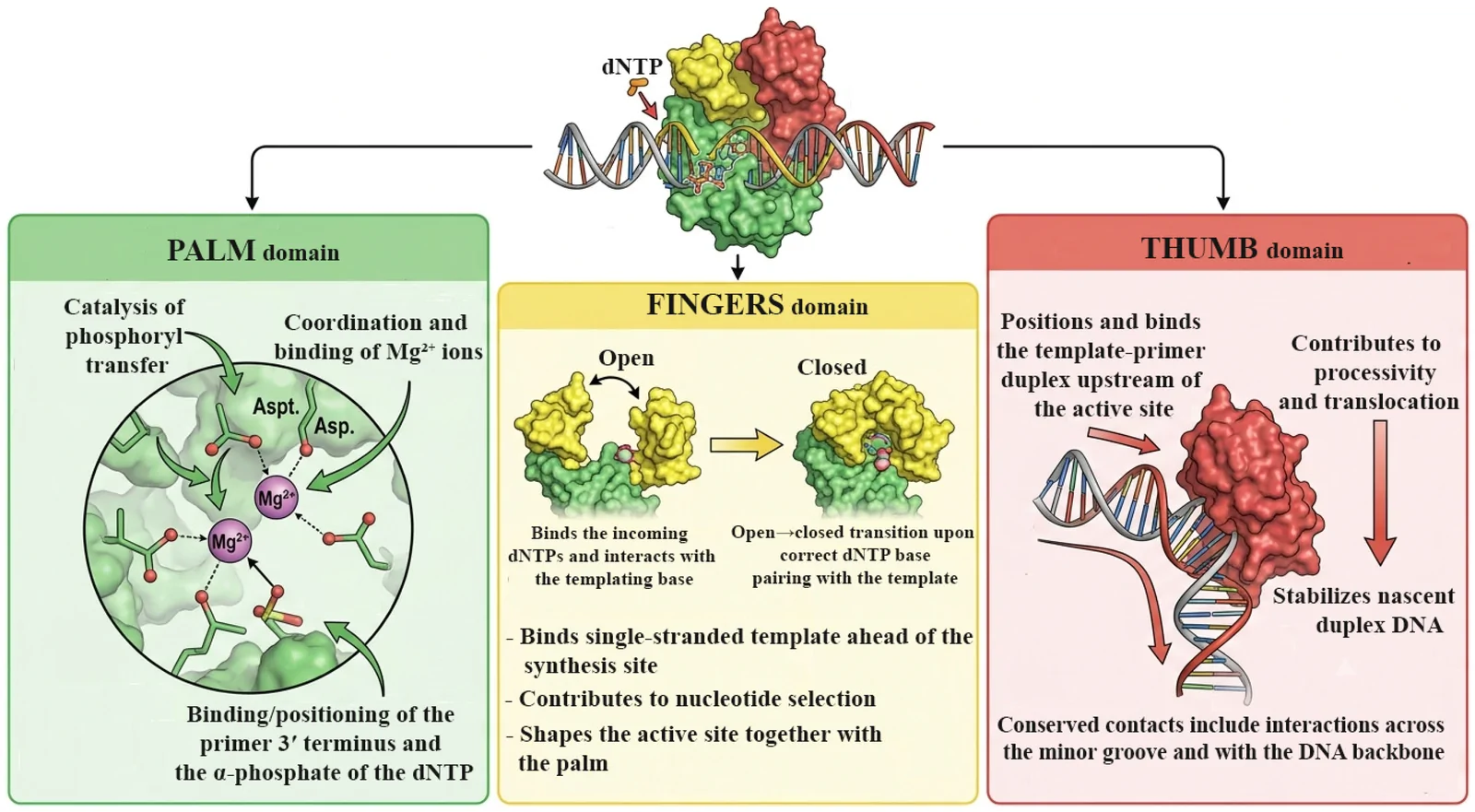
Structural and functional domains of a DNA polymerase. The crystal structure of *Pyrococcus furiosus* family B DNA polymerase (PDB ID: 2JGU).

Despite the considerable diversity of primary structures and the evolutionary origins of various DNA polymerase families, most enzymes share a similar structural organization of their catalytic core. The classic architecture of DNA polymerase is described by the “right hand” model, comprising three main structural domains: the palm, the fingers, and the thumb (Figure 2), which together form a cleft that binds the DNA template-primer complex and the incoming dNTPs [1,92,98,99,100].

Palm is the most conserved domain and contains the active site of the polymerase, which catalyzes the phosphoryl transfer reaction during nucleotide incorporation [98]. This domain contains conserved carboxylate groups that are clustered within the active site and plays an important role in catalysis by coordinating catalytic metal ions via a two-metal ion mechanism [92,100]. In addition to catalysis, the palm domain binds the primer terminus and the α-phosphate of the incoming dNTP, underscoring its role in the direct positioning of substrates for bond formation [92]. The functions mentioned above-such as phosphoryl transfer, coordination of metal ions, and binding/positioning of the 3′-end of the primer and the α-phosphate group of the dNTP-characterize a structurally conserved region of the polymerase across all families [92,98,99].

The finger domain participates in the formation of the active-site cleft wall and is responsible for key interactions with the incoming nucleoside triphosphate and nitrogenous base, which are incorporated into the growing chain [92,98]. Based on data from Family A polymerases, the finger domain can also bind to single-stranded DNA outside its active site and contains the conserved amino acid residues of motif B (including an invariant lysine present in all DNA-dependent polymerases), which are involved in binding and directing the nucleotide at the junction of the Palm and Finger domains [92]. In the absence of a substrate, the finger domain is in an open conformation and closes only when the incoming dNTP binds complementarily to the template [101]. Unlike polymerases of families A and B, repair polymerases (Families X and Y) have fewer contact points with the substrate, which is consistent with their lower strand synthesis accuracy [98,102,103].

The thumb domain holds the primer-template duplex in place by directly interacting with the double-stranded region of the DNA primer [92]. Across various polymerase families, the thumb domain contributes to the correct positioning of the DNA duplex and processivity. Structurally, the thumb stabilizes the DNA duplex by forming conserved binding sites with the sugar phosphate backbone of DNA and through the minor groove. The conserved loops of the thumb domain bind to the backbone and to the α-helix and mediate interactions through the minor groove of the DNA-primer duplex [98]. Several factors associated with the thumb domain can enhance polymerase processivity. For example, in T7 DNA polymerase, thioredoxin binds to a loop in the thumb domain, sterically and electrostatically preventing DNA dissociation during the catalytic cycle [100]. The architecture of the thumb domain varies among polymerase families; however, the function of retaining the DNA duplex remains conserved [98,100,102].

Although polymerases share a common architecture, polymerases from different families differ in their functional domains. Family A polymerases, represented by the Taq, Tth, and Bst enzymes, typically contain a N-terminal 5′→3′ exonuclease domain involved in the removal of RNA primers and DNA processing. In contrast, most Family B polymerases lack this activity but possess a 3′→5′ exonuclease domain that enables the correction of replication errors, thereby increasing the accuracy of DNA synthesis [17]. The presence of proofreading activity is one of the key reasons for the widespread use of Family B polymerases [69,70,72,73,74].

Structural studies have shown that the high thermal stability of archaeal polymerases is due to an increased number of ionic interactions and hydrophobic contacts, as well as more compact packing of the protein molecule compared to its mesophilic counterparts [1,2]. These features ensure the maintenance of catalytic activity at temperatures above 90 °C and make these enzymes particularly attractive for molecular biology and diagnostic applications.

Current protein engineering methods allow the modification of both the catalytic and supporting domains of polymerases, specifically altering their accuracy, processivity, resistance to inhibitors, and reverse transcription capability. These structural and functional characteristics of enzymes underlie most strategies for developing next-generation recombinant polymerases.

## 4. Expression and Purification of Recombinant DNA Polymerases

The development of recombinant DNA technology has made thermostable DNA polymerases more widely available, allowing their industrial production to meet the requirements of molecular biology, diagnostics, and biotechnology. Current approaches to polymerase production include choosing an optimal expression system, overcoming the challenges of heterologous protein synthesis, developing effective purification protocols, and ensuring quality control of the final enzyme product. The choice of a specific platform is determined by the protein yield, solubility, catalytic activity, and production costs [69,104,105,106].

### 4.1. Recombinant DNA Polymerase Expression Systems

*E. coli* cells are the most extensively used platform for producing thermostable DNA polymerases. The popularity of this system derives from its rapid growth rate, ease of genetic manipulation, low cost of cultivation, and the ability to scale the process up to an industrial level. The strains BL21(DE3), BL21(DE3)pLysS, BL21(DE3) RIPL, and Rosetta(DE3) are most commonly used for polymerase expression, as they ensure high levels of target protein synthesis under the control of the T7 promoter [13,107,108,109].

The BL21(DE3)pLysS strain also expresses T7 bacteriophage lysozyme, which suppresses the background activity of T7 RNA polymerase and reduces the risk of toxic effects of the recombinant protein on the host cell. BL21(DE3) RIPL and Rosetta(DE3) contain sets of rare tRNAs that compensate for differences in codon usage between *E. coli* and other organisms. This feature increases the expression success rate from 64% in BL21(DE3) to 77% in Rosetta(DE3), allowing for more efficient expression of many archaeal polymerases [110]. However, an increase in translation rate is not always accompanied by an increase in soluble protein yield, and in some cases, can lead to the formation of aggregates and inclusion bodies [111,112].

An alternative to bacterial systems is the yeast *Pichia pastoris*, which secretes recombinant enzymes into the culture medium and produces impurity-free preparations [113,114]. For example, the secreted variant of KOD DNA polymerase (RKOD) reached a yield of ~250 mg/L after 6 days of induction in flasks while maintaining high specific activity and accuracy. An additional advantage of this platform is its ability to perform post-translational modifications of proteins, such as N-glycosylation [115,116]. In this regard, it has been shown that when KOD polymerase is expressed in *P. pastoris*, glycosylation of the enzyme can have a positive effect on the thermal stability and performance characteristics [37,117]. However, the use of the methanol-inducible AOX1 promoter requires more complex process management and increases the duration of the production cycle.

Recently, cell-free protein synthesis (CFPS) systems have attracted increasing attention. These systems enable the production of recombinant polymerases within a few hours, without the use of living cells. This allows the process, from PCR product to finished activity-screened protein, to be completed in less than 24 h [118,119] with yields of up to 1200 mg/L [118,120]. However, a crucial issue is the stability of DNA templates (especially linear DNA molecules), which is compromised by rapid degradation by endogenous nucleases in cell-free extracts. To achieve yields comparable to those of plasmid DNA, it is necessary to introduce nuclease inhibitors (GamS) or modify DNA ends (capping), which complicates the production cycle [119,121]. Although crude cellular extracts are inexpensive, the presence of proteases and nucleases limits their use, whereas the PURE system, which ensures the absence of these impurities, is also costly and requires extensive optimization [121,122]. Such systems offer a high degree of flexibility in experimental design and are particularly valuable for screening mutant libraries and conducting the directed evolution of enzymes [118,120,121]. However, the high cost of CFPS currently limits its application mainly to research tasks and the rapid prototyping of new enzyme variants. CFPS systems are less cost-effective than classical microbial fermentation systems and are not yet considered a low-cost option for large-scale commercial enzyme production [118,121].

### 4.2. Issues of Archaea and Thermophilic Polymerase Expression in Mesophilic Hosts

The production of active thermostable polymerases in mesophilic organisms is often accompanied by several technical challenges. One of the main issues is the difference in codon usage between the thermophilic microorganisms and *E. coli* cells. The high GC content of genes, the presence of rare codons, and structural features of mRNA can significantly reduce translation efficiency and affect the overall protein yield [13,123]. Additional complications arise from the misfolding of recombinant proteins and formation of inclusion bodies. As most thermophilic polymerases have evolved to function at temperatures significantly higher than *E. coli*’s physiological growth temperature, the cellular folding systems of the mesophilic host do not always ensure the correct formation of the enzyme’s three-dimensional structure [13,124,125]. To overcome this difficulty, strategies such as lowering the induction temperature, co-expressing molecular chaperones, using solubilizing protein tags, and optimizing culture conditions have been employed [124,125,126]. The toxicity of certain polymerases toward host cells is also a significant limitation. A well-known example is Taq polymerase, whose constitutive expression under its native promoter is toxic to *E. coli*, necessitating the use of inducible expression systems [13]. These expression bottlenecks are systematically addressed using two approaches: pre-translational codon optimization and post-translational chaperone co-expression. While codon optimization directly resolves rare codon-induced translation stalling, premature termination, unwanted internal reinitiation/TGA stop sites, and low basal yield (as demonstrated with synthetic Pfu polymerase) [123], it cannot mitigate post-translational misfolding, temperature mismatches, or aggregation into inclusion bodies.

Conversely, chaperone co-expression directly addresses the folding environment, preventing inclusion body formation and promoting the solubility of large, multi-domain thermophilic enzymes—particularly when utilizing homologous or thermostable chaperones, such as *Thermus* DnaK, rather than host *E. coli* systems [126]. However, chaperone co-expression increases the metabolic load on the host cell and does not resolve sequence-level translational limitations. Consequently, maximizing the yield of active, full-length thermophilic and archaeal polymerases often requires a synergistic, multi-tiered approach combining both strategies. Taken together, these factors necessitate a comprehensive approach for expression optimization, including both rational gene design and the choice of an optimal expression platform.

### 4.3. Thermal Stability and First-Stage Purification

One of the most significant advantages of thermostable DNA polymerases is their ability to withstand high temperatures during the early stages of purification. After the cells of the recombinant producer are lysed, the cell lysate is heated to temperatures of 65–80 °C, which leads to the denaturation and precipitation of most host proteins, whereas the target polymerase retains its native structure and catalytic activity [13,37]. This procedure reduces the level of protein impurities before chromatographic purification is performed. A similar approach was successfully applied to the production of secreted polymerases in a *P. pastoris* system, where preliminary heating of the culture supernatant effectively removed yeast background proteins [37].

Owing to its simplicity of application and high efficiency, heat treatment has become one of the key steps in most processes for producing thermostable polymerases, both in research laboratories and industrial production.

### 4.4. Chromatographic Methods for the Purification of DNA Polymerases

Despite the effectiveness of heat treatment, one or more stages of chromatographic purification are typically required to obtain highly purified products. The most common method is immobilized metal affinity chromatography (IMAC), which is based on the interaction of polyhistidine tags (His-tags) with nickel or cobalt ions immobilized on a sorbent matrix. The use of His-tags allows the production of highly pure products after a single stage of affinity purification [105,106].

Methods such as ion-exchange chromatography, gel filtration, and hydrophobic chromatography have been used to remove impurities and improve the product quality. Heparin-affinity chromatography is particularly interesting because heparin structurally mimics nucleic acids and can effectively bind to DNA-binding proteins [127,128], including polymerases [129,130,131]. Owing to its high selectivity, this method has been extensively used to produce enzymes for PCR and sequencing.

The choice of purification scheme is determined by the characteristics of the specific polymerase and the requirements for the final product. In some cases, a single stage of IMAC purification is sufficient for research purposes; however, the production of diagnostic reagents requires additional purification and quality control steps.

### 4.5. Quality Control and Trends in DNA Polymerase Production Technologies

Modern diagnostic and biotechnological applications require high standards of enzyme product quality. In addition to their high specific activity, recombinant polymerases must be free of foreign nucleases, contain minimal levels of bacterial DNA and RNA, exhibit high storage stability, and demonstrate reproducible catalytic characteristics from batch to batch. Enzyme quality is assessed by electrophoretic analysis, specific activity determination, residual nuclease activity testing, thermal stability analysis, and amplification efficiency monitoring using standard DNA targets. For diagnostic enzymes, their resistance to PCR inhibitors and their performance in complex biological samples have additionally been evaluated [16,18].

Trace amounts of host bacterial DNA and residual nucleases from recombinant production are major risks in high-sensitivity PCR workflows. Commercial master mixes that are not treated can still harbor amplifiable contamination in up to 85% of samples (~10 copies per well), and commercial Taq products can carry between 10^2^ and 10^5^ host genome equivalents per unit [132,133,134,135]. To eliminate background contamination (~100 copies) without inducing false positives or negatives, it is essential to use matrix-tailored decontamination protocols [133,136]. Although enzymatic clearance utilizing DNase I effectively digests host DNA, it requires precise thermal and chemical inactivation (e.g., DTT at 80 °C for 30 min or 94–95 °C for ~50 min) to prevent PCR inhibition and template degradation downstream [133,134]. Alternatively, heat-labile, double-strand-specific nucleases (HL-dsDNase at 0.1 U/mL) can degrade >99% of >73 bp double-stranded contaminants while preserving single-stranded primers. This method only requires mild inactivation (50 °C for 15–20 min with DTT). However, the activity of this method is buffer-dependent and can drop tens- to hundreds-fold. This must be compensated for with Mg^2+^/Ca^2+^ supplementation [136]. Photochemical/chemical treatments (e.g., EMA for ~10 min, UV, psoralen) risk enzyme inactivation or narrow operational windows [136,137], whereas physical ultrafiltration provides a reliable, non-inhibitory alternative that removes up to ~100 contaminant copies without introducing residual nuclease activity [133].

Current trends in the development of polymerase production technologies are linked to a transition from the isolation of natural enzymes to the creation of specialized recombinant variants with specific properties. Producing enzymes with improved catalytic characteristics is a key factor driving the development of modern DNA polymerase engineering technologies and the creation of new commercial products.

## 5. Molecular Engineering of DNA Polymerases: Designing Enzymes with Customized Properties

Advances in molecular biology, NGS (next-generation sequencing), and molecular diagnostics have increased the requirement for DNA polymerases. In the early stages of PCR development, the primary criterion for enzyme selection was thermal stability, whereas modern enzymes require a combination of high synthesis accuracy, throughput, resistance to inhibitors, ability to work with modified nucleotides, and effective amplification of complex templates [2,15,17]. Consequently, most modern commercial enzymes are products of rational design, directed evolution, or domain engineering. The use of these approaches has led to the development of an entire generation of polymerases with improved characteristics that far surpass those of their natural counterparts.

### 5.1. Extension of the Substrate Specificity of DNA Polymerases

One of the most rapidly developing areas of molecular engineering is the creation of polymerases capable of effectively processing noncanonical substrates. Under normal conditions, most DNA polymerases strictly distinguish between deoxyribonucleotides and ribonucleotides due to a “steric gate” mechanism formed by conserved amino acid residues in the enzyme’s active site [8,9]. Mutations in the active-site region allow for changes in enzyme substrate specificity. The best-known example is the development of the Therminator polymerase based on the polymerase from *Thermococcus* sp. 9°N. Inactivation of 3′→5′ exonuclease activity through the D141A and E143A substitutions, in combination with the A485L mutation, resulted in an enzyme able to efficiently incorporate ribonucleotides, dideoxynucleotides, and a wide range of chemically modified nucleotide analogs [138].

Similar approaches have been applied to Vent and DeepVent polymerases. Substitutions A488L, Y499L, Y412V, and Y497S enhance the ability of these enzymes to incorporate modified substrates, making them promising tools for the synthesis of synthetic nucleic acids and various sequencing methods [139]. Other Family B polymerases, particularly KOD, possess high inherent thermal stability and structural flexibility, making them attractive starting scaffolds for further engineering. Owing to their high processivity and tolerance for active site mutations, these enzymes have become the backbone for the development of polymerases capable of synthesizing artificial nucleic acids (XNA), including HNA, FANA, CeNA, and TNA [138,140]. Such enzymes are regarded as important tools in synthetic biology and research on alternative forms of heredity.

### 5.2. Polymerases for Next-Generation Sequencing Technologies

All advances in sequencing-by-synthesis technologies have necessitated the development of enzymes capable of efficiently incorporating bulky fluorescently labeled nucleotides and reversible synthesis terminators. To solve this problem, specialized mutant variants of archaeal polymerases have been developed. One of the most extensively studied examples is the KOD Mut_C2 variant, which contains a combination of substitutions D141A, E143A, L408I, Y409A, and A485E. The introduction of these mutations enhanced the enzyme’s ability to incorporate modified nucleotides (e.g., 3′-O-azidomethyl-dATP, labeled with Cy3 dye), which are used in modern sequencing technologies [138]. Further optimization led to the generation of the KOD Mut_E10 variant, which contains an even broader set of mutations (D141A, E143A, L408I, Y409A, A485E, S383T, Y384F, V389I, V589H, T676K, and V680M) and is characterized by a higher efficiency in incorporating bulky fluorescent nucleotide analogs. These enzymes have become an important part of modern high-throughput sequencing platforms, ensuring accurate and controlled DNA synthesis through cyclic addition of labeled nucleotides [141].

### 5.3. Improving the Processivity of DNA Polymerases

In addition to modifying substrate specificity, one of the most important goals in enzyme engineering is increasing enzyme processivity. High processivity allows polymerases to synthesize long DNA fragments without dissociating from the template, which is particularly important for amplifying long genomic regions and preparing libraries for sequencing. A well-known example of this approach is the development of Phusion. The construct consisted of the Pfu polymerase fused to the DNA-binding protein Sso7d from the archaeon *Sulfolobus solfataricus* [142]. The incorporation of the Sso7d domain increases the affinity of the enzyme for the DNA template, boosts the rate of synthesis, and improves the efficiency of amplifying complex GC-rich DNA regions while maintaining high replication fidelity [1,142]. A similar principle has been used to develop several other hybrid enzymes containing additional DNA-binding domains, single-stranded DNA-binding proteins, or modified template-interaction regions. The work [143] describes the generation of a mutant polymerase based on the Tkod polymerase from *Thermococcus kodakarensis* by replacing its domains with analogous ones from Pfu; the resulting multitope chimeric mutant successfully combined high speed, processivity, thermal stability, and accuracy. The intervening domain of Taq polymerase was replaced with a homologous region of the proofreading domain from Pol I (*E. coli*), resulting in Taq polymerase acquiring additional 3′→5′ exonuclease activity, which enables it to correct errors [144]. Domain engineering is currently considered one of the most effective tools for improving polymerase performance.

### 5.4. Increased Resistance to PCR Inhibitors

A key challenge in molecular diagnostics is the presence of substances in clinical and environmental samples that inhibit DNA polymerase activity, including hemoglobin, lactoferrin, immunoglobulins, heparin, salts, bile acids, urea, humic acids, and other compounds commonly found in soil samples [16,18].

Directed evolution and rational mutagenesis have been used to increase resistance to inhibitors. Modern variants of Taq DNA polymerase can directly amplify DNA from whole blood, saliva, and several other biological materials without prior purification of nucleic acids. These enzymes form the basis of Direct PCR technology and have significantly simplified diagnostic procedures [16]. One example is a study [145] in which the halophilic polymerase BR3 Pol was obtained. It possesses natural salt tolerance due to an excess of negatively charged amino acids and flexible, disordered loops in its structure. Based on a homologous region of this polymerase, chimeric polymerases were produced in which homologous regions—such as the exonuclease domain and the “thumb” domain-were replaced with corresponding regions from BR3 Pol. As a result, the resulting chimeric variants, KOD^BR3exo^ and KOD^BR3thumb^, exhibited an expanded range of salt tolerance (up to 0.3 M NaCl), which is critical for modern technologies such as nanopore sequencing, where buffer solutions with high ionic strengths are required.

In addition to the production and application of chimeric polymerases in standard PCR procedures [146,147,148,149,150], there is a need to obtain enzymes with improved properties for alternative amplification methods such as LAMP and LIMA (linear target isothermal multiple amplification) [29,151]. LAMP is a widely used method in clinical diagnostics, and the most common polymerase used in LAMP is Bst polymerase [61,152,153,154]. Major commercial suppliers, such as NEB, Thermo Fisher, and Sigma, offer their own lines of Bst polymerases (Bst 2.0 and Bst 3.0). However, these commercial products cannot always meet the demands of certain applications, such as those involving inhibitors in the samples [155]. Molecular engineering allows the creation of chimeric Bst polymerases that combine increased thermal stability, resistance to inhibitors, and improved amplification efficiencies. For example, the Br512 fusion polymerase, which contains the positively charged HP47 domain and additional point mutations, retained its activity at 74 °C in the presence of 1 M urea, allowing the LAMP assay to be completed in less than 10 min [156]. Similarly, the introduction of the K431D and K431E mutations, along with the DNA-binding domain of the DNA ligase from the archaeon *Pyrococcus abyssi*, into the Bst polymerase enabled the enzyme to retain its activity at 70 °C for 8 h, increased resistance to inhibitors, and enabled the detection of *Salmonella typhimurium* at concentrations of 10^2^ ag/μL or 100 CFU/mL [154]. Further combination of the Sto7d and HP47 domains in the chimeric polymerase HpStBL increased the sensitivity of the assay for virus-infected whole blood to 93.75% and ensured effective performance of RT-LAMP reactions [157].

### 5.5. Hot-Start Technology

Efforts to reduce the level of nonspecific amplification and primer dimer formation have led to the development of hot-start polymerases, which are widely used in modern PCR technologies [158,159]. Hot-start technologies are designed to temporarily inhibit the activity of DNA polymerase during the preparation of the reaction mixture and activate it only after high-temperature heating, thereby reducing primer dimer formation and nonspecific amplification [147,158,160,161]. Three main strategies are used in hot-start technologies: the reversible chemical modification of polymerases, thermolabile inhibitory antibodies, and aptameric inhibitors. Chemical hot-start is based on the reversible covalent blocking of the functional groups of the enzyme. For example, citraconylation of the lysine amino groups of Taq polymerase blocks enzyme activity; activity is regained after high-temperature incubation due to diacylation, caused by a decrease in the pH of Tris buffer upon heating [162]. Commercially available products such as AmpliTaq Gold and HotStart Taq generally require activation for 5–15 min at 95 °C; however, prolonged heating can lead to depurination and damage to the template DNA [163]. Hot-start antibody systems are based on the binding of thermolabile antibodies to the active site or functionally important domains of the polymerase, which provides inhibition at low temperatures and subsequent dissociation after denaturation of the antibodies at 90–95 °C [161,163,164,165]. Such systems are characterized by rapid activation; however, a significant molar excess of antibodies is often required for effective activity suppression, and complete inhibition is not always achieved [163,164].

An alternative approach involves selectively screening aptamer inhibitor single-stranded oligonucleotides using the SELEX method for binding to the polymerase at low temperatures and dissociating upon heating [161,166]. For Taq polymerase, aptamers were obtained that effectively inhibit the enzyme at 20–25 °C and thermally reversibly release it at temperatures above 40 °C, which increases the sensitivity of the assay and reduces the formation of nonspecific products [166,167]. However, the effectiveness of this approach depends on the precise selection of aptamer concentration and the properties of the specific polymerase.

In general, chemical modifications provide the strongest inhibition, antibody-based systems are characterized by rapid activation, and aptamers are distinguished by their high stability and flexibility across a range of reaction conditions, making all three strategies valuable tools for the development of modern hot-start DNA polymerases.

### 5.6. Development of Hybrid and Multifunctional DNA Polymerases

Current trends in molecular engineering are focused on creating multifunctional enzymes that combine several useful properties into a single molecule. Examples of such enzymes include Phusion, Q5, PfuUltra, PrimeSTAR, KOD FX, and other high-fidelity next-generation commercial enzymes. Polymerases with additional reverse transcriptase activities are of particular interest. Previously, reverse transcriptase activity was demonstrated in Tth polymerase in the presence of Mn^2+^ ions [168]. The main limitations of Tth as a reverse transcriptase are its dependence on Mn^2+^, reduced synthesis fidelity, and relatively low efficiency in copying long and structured RNA templates [169]. The best-known example of a modified enzyme is RTX, an engineered variant of an archaeal polymerase of the Family B capable of efficiently utilizing both DNA and RNA templates while retaining 3′→5′ proofreading activity [170]. The emergence of such enzymes eliminates the need for separate reverse transcriptase and DNA polymerase enzymes, opening prospects for streamlined single-enzyme RT-PCR and other RNA analysis workflows.

Thus, current approaches to DNA polymerase engineering are gradually shifting from modifying individual properties to creating multifunctional enzymatic platforms that combine high synthetic accuracy, throughput, resistance to inhibitors, and expanded substrate specificity. These enzymes form the foundation of most of the current technologies in molecular biology, sequencing, and diagnostics.

## 6. Recombinant DNA Polymerase Applications in Biotechnology, Diagnostics, and Genomic Research

Recombinant DNA polymerases are among the most widely used enzymes in modern biotechnology. Their applications span a wide range of tasks, from routine DNA amplification to high-throughput genome sequencing and the development of molecular diagnostic systems. Advances in recombinant expression and molecular engineering technologies have enabled the production of enzymes with improved thermal stability, enhanced synthesis fidelity, inhibitor resistance, and expanded substrate specificity, thereby broadening their range of practical applications [1,2,15].

### 6.1. Polymerase Chain Reaction and Its Modifications

The most widely known application of thermostable DNA polymerases is in PCR. Since the discovery of Taq polymerase from *Thermus aquaticus*, PCR has become a key tool in molecular biology and diagnostics. The high thermal stability of this enzyme eliminated the need to add fresh polymerase after each amplification cycle and allowed for automation of the process [13,14]. Currently, various types of polymerases with different properties are used for different PCR variants; however, Taq polymerase remains the gold standard for routine diagnostics and the analysis of large numbers of samples because of its high synthesis rate and low cost. For applications demanding minimal amplification errors, high-precision enzymes from Family B, Pfu, Vent, Deep Vent, and their modified variants, such as Phusion and Q5, are used. The presence of 3′→5′ exonuclease activity allows these enzymes to achieve a significantly lower error rate compared to Taq polymerase [1,21].

In addition to conventional PCR, recombinant polymerases are widely used in quantitative PCR (qPCR), digital PCR (dPCR), multiplex PCR, and various forms of long-range PCR, designed to amplify long genomic regions. For such tasks, enzyme throughput, resistance to inhibitors, and the ability to work effectively with GC-rich templates are of particular importance [18].

Developments in molecular engineering have led to the emergence of numerous commercial DNA polymerases optimized for specific tasks ranging from routine PCR and diagnostics to high-precision cloning, long-range PCR, and next-generation sequencing. The properties of the most used commercial enzymes are listed in Table 2.

As shown in Table 2, current commercial polymerases differ in terms of synthesis accuracy, throughput, the presence of 3′→5′ and 5′→3′ exonuclease activity, and tolerance to complex templates, which determines their application in various fields of molecular diagnostics and genomic research.

### 6.2. Molecular Diagnostics of Infectious and Hereditary Diseases

One field of application of recombinant polymerases is molecular diagnostics, where PCR technologies are used to detect bacterial, viral, and parasitic pathogens, diagnose hereditary diseases, analyze cancer mutations, and monitor the effectiveness of therapy. Current diagnostic test systems must be capable of processing complex biological samples, such as blood, sputum, feces, urine, and saliva, which often contain amplification inhibitors. Under such conditions, inhibitor-resistant polymerase variants and specialized buffer systems become crucial [16,17,18].

The development of Direct PCR technologies has made it possible to perform amplification directly from unpurified samples without preliminary DNA extraction, which significantly reduces analysis time and lowers the risk of contamination [171,172]. This approach is particularly valuable for rapid diagnosis of infectious diseases and large-scale screening studies [173]. Hot-start polymerases are also important because they reduce nonspecific amplification and primer dimer formation [174]. This leads to increased sensitivity and specificity of the diagnostic tests, particularly in multiplex formats [175].

### 6.3. DNA Sequencing and Genomic Research

DNA polymerases play a crucial role in NGS technologies [176]. Nearly all modern sequencing platforms, with the exception of certain ligation-based methods, rely on enzymatic DNA synthesis both during library preparation and during the nucleotide sequencing process, making the properties of polymerases one of the main factors determining the accuracy and throughput of the analysis [177]. Engineered variants of Family B polymerases capable of efficiently incorporating fluorescently labeled nucleotides and reversible synthesis terminators are particularly important. Such enzymes form the basis of sequencing-by-synthesis technologies used in modern high-throughput sequencing systems [176,178]. High-fidelity polymerases are also used to prepare libraries for genome, metagenome, and transcriptome sequencing [177]. A low amplification error rate is critical for analyzing rare variants, single-nucleotide polymorphisms, and somatic mutations [179].

### 6.4. Synthetic Biology and Genetic Engineering

In synthetic biology, recombinant polymerases are used for gene cloning, assembly of genetic constructs, site-directed mutagenesis, and creation of synthetic genomes [180,181]. High-fidelity enzymes of Family B decrease the probability of random mutations during the amplification and assembly of large DNA constructs [179]. Engineered variants of polymerases that can function with modified nucleotides and XNA are of particular interest. Such enzymes provide opportunities for developing alternative genetic systems, creating new biocatalysts, and expanding the chemical diversity of nucleic acids [138,140]. The development of Therminator-like enzymes, KOD mutants, and other specialized polymerases has enabled effective use of fluorescently labeled, chemically modified, and reversibly blocked nucleotides in various biotechnological applications [138,182].

### 6.5. Isothermal Amplification Methods

In addition to PCR, recombinant polymerases are also widely used for isothermal amplification. The best-known example is the LAMP method, which uses Family A polymerases with pronounced strand displacement activity, such as Bst polymerase. The development of engineered variants of Bst 2.0 and Bst 3.0 has further expanded the capabilities of isothermal diagnostics and target RNA analysis [60,183]. Isothermal methods allow amplification without thermal cycling and are becoming an important basis for portable diagnostic platforms designed for use outside specialized laboratories. High reaction rates and resistance to inhibitors make these systems promising for veterinary diagnostics, food safety, and environmental monitoring [60,183].

Rolling circle amplification (RCA) is an isothermal technique wherein a strand-displacing DNA polymerase continuously extends a primer around a circular ssDNA template, generating a concatemeric single-stranded product with tandem repeats [184,185,186]. Phi29 polymerase (Family B) is widely used in RCA because it combines processive DNA synthesis with strand displacement. It also has 3′→5′ exonuclease activity, which can affect primer design and has been deliberately exploited to process protruding 3′ target tails in specialized assays. Bst polymerase can also support some RCA formats [185,187].

Rolling circle amplification (RCA) is a versatile isothermal technique that delivers high target specificity and robust signal amplification. This is achieved through the generation of long, repetitive single-stranded concatemers. This enables diverse applications, ranging from in situ single-molecule imaging to preparative plasmid amplification, across DNA and RNA targets [184,185,186,187]. However, there are distinct technical limitations to its practical deployment: padlock-dependent formats are constrained by ligation efficiency and target accessibility, and linear RCA is less sensitive than exponential variants, which carry a higher risk of non-specific background noise. Furthermore, the physical transport of high-molecular-weight concatemers can be hindered in gels and microfluidic channels. Additionally, the technique is highly vulnerable to carryover contamination, necessitating strict workflow segregation and platform-specific validation [184,185,186].

**Figure 3:**
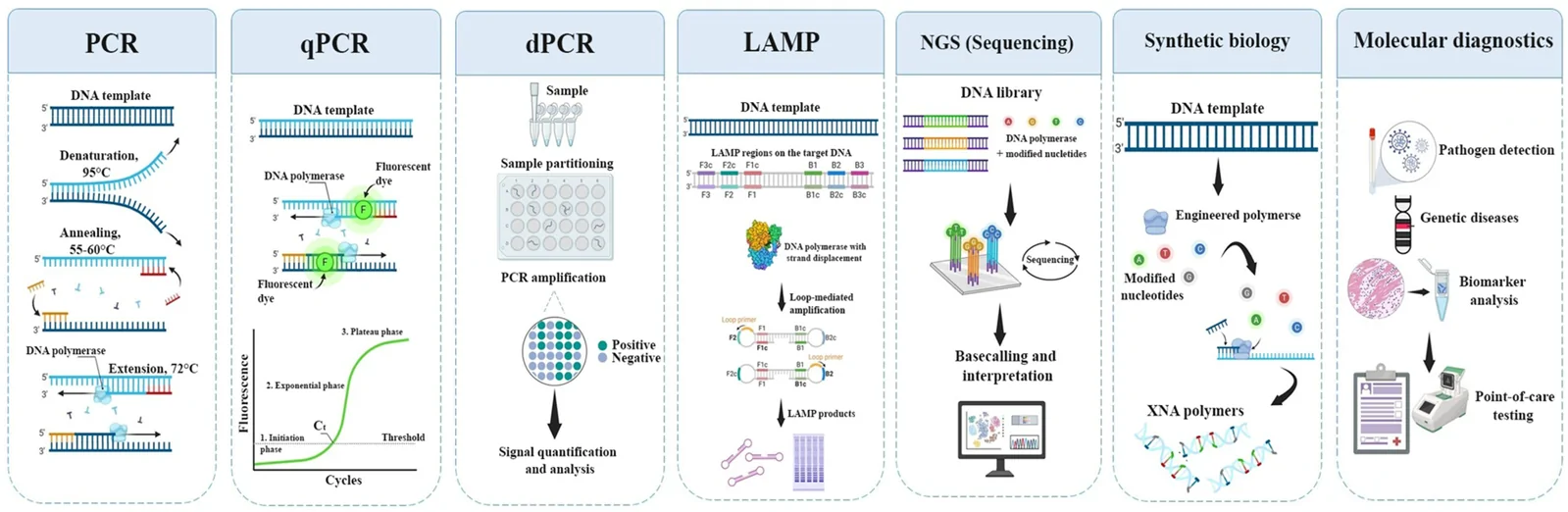
Applications of recombinant DNA polymerases in molecular biology.

RCA has been used for pathogen and viral detection, antimicrobial-resistance genes, SNP and mutation genotyping, miRNA and other RNA assays, telomerase and protein-linked biosensing, in situ molecular imaging, digital counting, lateral-flow diagnostics, sequencing/cloning workflows, and amplification of plasmids or circular viral genomes. Published examples include assays for Ebola, SARS-CoV-2, Zika, dengue, beta-lactamase resistance genes, and targets embedded in *Cryptococcus neoformans* genomic DNA [188,189,190]. Figure 3 illustrates a comprehensive compilation of DNA polymerase-driven nucleic acid assays, highlighting their activity and their operational frameworks.

### 6.6. DNA Polymerases in PCR-Based Whole-Genome Amplification Methods

PCR-based whole-genome amplification (WGA) strategies include degenerate/random-primed protocols, quasi-linear systems, and adapter-ligation approaches. Classical degenerate oligonucleotide-primed PCR (DOP-PCR) and primer extension preamplification (PEP-PCR) utilize degenerate primers (e.g., 15mers in PEP) to generate amplicons that typically range from a few hundred base pairs to several kilobases [188,189]. Conventional DOP-PCR uses low-stringency initial cycles (~25–30 °C), driven by Taq DNA polymerase. Modified protocols, such as LL-DOP-PCR, dcDOP-PCR, mDOP-PCR, and iDOP-PCR, introduce proofreading thermophilic enzymes, like Pwo polymerase, to increase product length, genome coverage, and sequence accuracy [188,189,190]. To minimize the propagation of early priming bias, quasi-linear techniques such as MALBAC and PicoPLEX/SurePlex decouple the preamplification stage from the exponential stage. MALBAC uses a thermostable strand-displacing enzyme, typically Bst DNA polymerase at ~65 °C, to generate linear copies before exponential cycling with Taq or Deep Vent (exo−) [189,190,191,192]. Alternatively, ligation-mediated methods (e.g., OmniPlex, GenomePlex, and Ampli1) chemically, enzymatically, or mechanically fragment the genome, attach defined adapters, and exponentially amplify targets via adapter-specific primers [188,192,193].

The choice of polymerase underlying the process fundamentally dictates WGA error rates, processivity, and structural uniformity. Non-proofreading enzymes, such as Taq (1–2 × 10^−4^ errors/nt) and Bst (1.5 × 10^−5^ errors/nt), introduce synthetic errors early on that exponentially propagate, which complicates the detection of low-frequency single-nucleotide variants (SNVs) [192,194]. Conversely, proofreading polymerases like Pwo have significantly higher fidelity (approximately 2.4 × 10^−6^ errors/nt), but they lack the strong strand-displacement activity required for quasi-linear displacement protocols [192]. PCR-WGA exhibits inherent representation bias and sensitivity to GC content, as demonstrated in 25 ng benchmark tests. In these tests, PEP achieved a 120-fold yield, covering 35.8% of high-GC genomes and 94.4% of low-GC genomes. In contrast, DOP achieved a 92-fold yield, covering only 9.7% of high-GC genomes and 17.0% of low-GC genomes. Despite these limitations, PCR-WGA’s thermocycling logic produces relatively even copy-number profiles, which are advantageous for copy number variation (CNV) and aneuploidy screening [189,193,195]. Furthermore, PCR-based approaches tolerate fragmented or damaged DNA templates far better than non-PCR isothermal methods. However, irreversible cleavage during ligation-mediated fragmentation poses a risk of losing single-copy alleles in single-cell setups [188,193].

For high-fidelity and long-read requirements, isothermal multiple displacement amplification (MDA) traditionally relies on the highly processive, mesophilic Phi29 DNA polymerase for proofreading. This yields products ranging from several to hundreds of kilobases with superior SNV fidelity and genome recovery [189,192]. However, classical Phi29-MDA is susceptible to stochastic priming bias, chimera formation, and poor performance with damaged inputs. To overcome these limitations, modern engineered thermostable Phi29 variants that operate at elevated temperatures (40–45 °C) have become commercially available (Thermo Fisher Scientific #A39390 and NEB #M0572). These variants resolve secondary structure impediments while retaining the extraordinary fidelity and processivity characteristic of isothermal displacement amplification.

Therefore, the central distinction is not simply “PCR versus MDA”. Within PCR-WGA, Taq favors robust, inexpensive cycling; Pwo favors fidelity; Bst supplies strand displacement for quasi-linear preamplification; and proprietary blends trade transparency for an optimized workflow. Primer architecture and amplification topology are at least as important as polymerase identity in determining coverage bias and allelic dropout.

### 6.7. Prospects for the Use of Recombinant Polymerases

Current trends in biotechnological development focus on the development of multifunctional enzymes that combine high synthetic fidelity, enhanced processivity, inhibitor resistance, and the ability to work with modified substrates. Special attention has been paid to polymerases that integrate DNA polymerase and RT capabilities, alongside enzymes tailored for use in artificial genetic systems and synthetic biology. Further development of rational design methods, directed evolution, and machine learning is expected to enable the creation of enzymes that are optimally adapted for specific diagnostic and research applications. This will make recombinant polymerases key tools in future genomic and biotechnological platforms.

Advances in AI (Artificial Intelligence) and ML (Machine Learning) have led to significant changes in approaches to protein design and analysis, the identification of evolutionary patterns, and the prediction of spatial structures [196,197]. One of the most significant achievements in recent years has been the development of the AlphaFold2 system by DeepMind, which has demonstrated high accuracy in predicting the three-dimensional structure of proteins [198]. Further development of the technology in the form of AlphaFold3 has significantly expanded the capabilities for modeling protein-DNA, protein-RNA, and protein-ligand complexes, which is particularly important in the design of new polymerase variants [199]. In addition to predicting spatial structure, large protein language models (PLMs), trained on hundreds of millions of protein sequences, have been actively developed in recent years [200,201].

AI-assisted DNA-polymerase engineering has moved beyond purely computational ranking: prospective wet-lab studies now show improved Bst and phi29 variants. The strongest result is MutCompute-guided Bst engineering, which yielded an additive triple variant operating rapidly at 73 °C with a 2.6 °C melting-temperature gain over Bst-LF [202]. Few-Shot Learning for Protein Fitness Prediction (FSFP) then demonstrated that only 20 target-specific measurements can improve Protein language models (PLM) selection of thermostable phi29 mutants [203], while ProtREM suggests that retrieval-enhanced multimodal models may also support zero-shot polymerase design [204].

Nevertheless, the field remains at an early proof-of-concept stage. Current evidence supports AI-assisted mutation prioritization for thermostability, not yet end-to-end design of broadly superior polymerases. The decisive next step is multi-objective, epistasis-aware engineering with prospective validation of fidelity, processivity, amplification bias, inhibitor resistance, and manufacturability.

## 7. Conclusions

Recombinant DNA polymerases constitute a key class of enzymes used in molecular biology, biotechnology, and diagnostics. Advances in genomic technologies, protein engineering, and synthetic biology have expanded the functional capabilities of these enzymes, transforming them from natural components of the cellular replication apparatus into highly specialized tools that can be used to perform a wide range of applied tasks.

DNA polymerase classifications reflect their evolutionary origins, structural organization, and functional specialization. Thermostable members of Families A and B have gained great practical significance because of their high thermal stability, processivity, synthetic accuracy, and adaptability to various molecular biology platforms. Family A polymerases, including Taq, Tth, and Bst, are the basis of classical PCR, quantitative PCR, and isothermal amplification methods, whereas archaeal Family B polymerases, such as Pfu, Vent, Deep Vent, KOD, and their derivatives, are used in high-fidelity amplification, cloning, and library preparation for next-generation sequencing.

Modern technologies for recombinant expression and purification have facilitated the industrial production of highly active enzyme products, and rational design, directed evolution, and domain engineering have made it possible to create polymerases with improved features. This results in enzymes with increased processivity, expanded substrate specificity, resistance to inhibitors, hot-start properties, and the ability to perform reverse transcription. These multifunctional systems underlie most of our current diagnostic and genomic technologies.

Further developments in this field will involve the integration of structural biology, computational modeling, artificial intelligence, and high-throughput screening of protein libraries. These approaches will enable the creation of next-generation DNA polymerases that combine high accuracy, synthesis speed, inhibitor resistance, the ability to handle modified nucleotides, and efficient performance in complex biological samples.

It is clear that recombinant thermostable DNA polymerases serve as the fundamental foundation for modern molecular biology. Further engineering optimization and functional diversification will enable the development of molecular diagnostics, genomic research, synthetic biology, and precision biotechnology, opening new opportunities for the creation of highly effective analytical and therapeutic platforms.

## Figures and Tables

**Table 1:** Classification of DNA polymerase families and their main features.

Family	Biological Functions	Taxonomic Distribution	Proofreading Activity (3′→5′ Exonuclease)	Primary Members	Thermostable Members	Biotechnological Applications	References
A	Replication, repair, and processing of Okazaki fragments	Bacteria, mitochondria, bacteriophages	No	*E. coli* Pol I, Taq, Tth, Bst	Taq, Tth, Bst	PCR, qPCR, LAMP, diagnostics	[25,26,27,28,29,30]
B	Genome replication, high-precision DNA synthesis	Archaea, eukaryotes, viruses	Yes	Pfu, Vent, Deep Vent, KOD, 9°N, Tgo	Pfu, Vent, Deep Vent, KOD, 9°N, Tgo	High-fidelity PCR, cloning, NGS	[31,32,33,34,35,36,37]
C	Major bacterial replicases	Bacteria	Yes	Pol IIIα (DnaE), PolC	None of commercial significance	Fundamental research	[38,39,40,41]
D	Replication of the archaeal genome	Archaea	Yes	PolD	Have been studied to a limited extent	It is being actively studied as an alternative to Family B polymerases for the development of ultra-precise and fast enzymes for NGS.	[22,42,43,44]
X	DNA Repair, BER, NHEJ	Eukaryotes/Prokaryotes	No (except for Polμ)	Polβ, Polλ, Polμ, TdT	PolX (TtPolX) *from Thermus thermophilus* or *Deicoccus radiodurans*	Used in biotechnology for gap-filling at high temperatures.	[45,46,47,48,49,50]
Y	Translesional synthesis	Bacteria, archaea, eukaryotes	No	DinB, Polη, Polι, Rev1	Dbh and Dpo4	Dpo4 has been commercialized and is used in specific sequencing and PCR methods to overcome severe template damage.	[51,52,53,54,55]
RT	DNA synthesis on an RNA template	Retroviruses, retrotransposons, telomerases, bacteria	No	MMLV RT, AMV RT, HIV-1 RT	RTX, TGIRT, GsI-IIC RT	RT-PCR, RNA-seq, diagnosis of RNA viruses	[56,57,58]

**Table 2:** Commercial thermostable DNA polymerases: origin, catalytic properties, and main applications.

Name	Origin	Family	Strand Displacement	Processivity	3′→5′ Proofreading	5′→3′ Exonuclease	Fidelity to Taq	Amplicon Length	Half-Life at 95 °C/100 °C (Hours)	Brand	Error Rate	Application
Taq	*Thermus aquaticus*	A	Weak	Low (~20–40 nt)	No	Yes	1×	3–8.1 kb	0.9/<0.1	New England Biolabs (NEB)/Thermo Fisher	~285 × 10^−6^ bases (NEB)	- PCR - Primer - Extension - DHPLC - Microarray - Analysis - Colony PCR
Tth	*Thermus thermophilus*	A	Weak	Low (~20–40 nt)	No	Yes	1×	Up to 3–5 kb	2–3/<0.3	Roche/Sigma-Aldrich	1–2 × 10^−5^	- PCR - to label DNA fragments with either radiolabeled nucleotides - to efficiently transcribe RNA targets into cDNA - for real-time PCR
Platinum Taq	*Thermus aquaticus*	A	Weak	Low (~20–40 nt)	No	Yes	1×	Up to 5 kb	~0.6–0.8/<0.08	Thermo Fisher	1–2 × 10^−5^	- Amplification of DNA (genomic, viral, and plasmid) - Hot-start PCR - RT-PCR
Platinum SuperFi II	Archaea of the genus *Pyrococcus*	B	No	High	Yes	No	>300×	Up to 20 kb	>3 at 95 °C	Thermo Fisher	~1 × 10^−7^	- High-fidelity PCR - Site-directed mutagenesis - Template generation for sequencing
Pfu	*Pyrococcus furiosus*	B	No	Low (<20 nt)	Yes	No	~10×	Up to 2–3 kb	~4–5/~0.5–1	Promega/Thermo Fisher	~1.3–2.0 × 10^−6^	- PCR - Primer extension reactions that require high-fidelity synthesis.
Vent	*Thermococcus litoralis*	B	Yes, temp. dependent	Average	Yes	No	5×	13.2 kb	6.7/1.8	NEB	<57 × 10^−6^ bases	- Primer extension - PCR
Deep Vent	*Pyrococcus* sp. GB-D	B	Yes, temp. dependent	Average	Yes	No	6×	14.0 kb	23/8	NEB	~ 1.0 × 10^−6^	- Primer extension - PCR
Therminator	*Thermococcus* sp. 9°N (D141A/E143A/A485L)	B	Yes	Average	No	No	1× (Low)	Up to 2–3 kb	~2–3 at 95 °C	NEB	Not for a PCR	- DNA sequencing by partial ribosubstitution - DNA sequencing using dideoxy or acyclo chain terminators - SNP analysis with dideoxy or acyclo chain terminators
Phusion	*Pyrococcus furiosus*	B	No	High	Yes (high)	No	50×	20 kb simple, 10 kb complex	>2–3/~0.5	NEB/Thermo Fisher	0.44 × 10^−6^	- PCR - Cloning - Long or Difficult Amplification - High-Throughput PCR
Q5U Hot-Start	Archaea of the genus *Pyrococcus*	B	No	High	Yes	No	>280×	Up to 20 kb	>2	NEB	~5 × 10^−7^	- Amplification of bisulfite-converted, deaminated, or damaged DNA
PrimeSTAR	Archaea of the genus *Pyrococcus*	B	No	High	Yes (high)	No	~40×	Up to 8.5 kb (plasmids up to 15 kb)	>4–5/~1.5	Takara Bio	~3.0–4.0 × 10^−7^	- High-fidelity PCR - Amplification of cDNA libraries - cDNA cloning for protein expression - Site-directed mutagenesis and mutant genotyping
KOD	*Pyrococcus kodakaraensis*	B	No	Very high	Yes (high)	No	~50–80×	Up to 6–10 kb (KOD FX up to 24 kb)	~12/~3	Toyobo/Merck (Millipore)	~2.5 × 10^−7^	- gDNA amplification for Sanger sequencing. - gDNA amplification to screen single-cell clones.
PfuUltra	*Pyrococcus furiosus*	B	No	Average	Yes	No	~18–20×	Up to 10 kb	>4–5/~1	Agilent Technologies	~5.0 × 10^−7^	- High-fidelity PCR - Generation of PCR products for blunt cloning - Site-directed mutagenesis
LongAmp	Unique blend of Taq and Deep Vent^®®^ DNA Polymerases	A + B	weak	Increased	Yes	Yes	2×	40 kb	~0.6–0.8/<0,08	NEB	~7.0 × 10^−6^	- Long-Range PCR - Colony PCR
Terra PCR	Novel non-Taq DNA polymerase		Weak	High	No	Yes	1–2×	Up to 2–4 kb	High in crude extracts	Takara Bio	~1.0 × 10^−5^	- Genotyping - Direct PCR from crude extracts, tissues, blood, FFPE samples and many more
OneTaq	Optimized blend of Taq and Deep Vent DNA polymerases	A + B	Weak	Increased	Yes	Yes	2×	20 kb	~0.6–0.8/<0.08	NEB	<140 × 10^−6^ bases	- High-Sensitivity PCR - High-Throughput PCR - Routine PCR - GC-rich PCR - AT-rich PCR - Colony PCR - Long PCR (up to ~6 kb genomic)
DreamTaq	*Thermus aquaticus*	A	Weak	Increased	No	Yes	1×	up to 6 kb from genomic DNA and up to 20 kb from viral DNA	~0.6–0.8/<0.08	Thermo Fisher	~1.1 × 10^−5^	- PCR amplification of DNA fragments - High-throughput PCR - Genotyping
Aztaq	*Thermus aquaticus*	A	No	High	No	Yes	1×	Up to 6 kb	~0.7–0.75	ArcticZymes Technologies	~2 × 10^−5^	-PCR/qPCR
ReverHotTaq	Optimized blend of Taq and Bst polymerases	A	No	High	No	Yes	1×	Up to 5 kb	0.7	Bioron GmbH	~2 × 10^−5^	- End-Point RT-PCR - Real-Time RT-qPCR
MegaFi Pro	Archaea of the genus *Pyrococcus*	B	No	High	Yes	Yes	>2000×	Up to 20 kb	>2	Applied Biological Materials	~6.6 × 10^−8^	- Ultra high-fidelity PCR - Next-Generation Sequencing - Accurate CRISPR Edit Detection - Mutagenesis
Phi29	Bacteriophage phi29	B	Yes	Very high (50–100 nt/s)	Yes	No	>100×	>70 kb (concatemers)	Inactivation at 65 °C	NEB	~1 × 10^−6^	Replication requiring a high degree of strand displacement
GspSSD LF	*Geobacillus* sp.	A	Yes	Very high (100 nt/s)	No	No	1–2×	10–50 kb (concatemers)	Inactivated at >80 °C	OptiGene	~2 × 10^−5^	LAMP and RT-LAMP
KAPA2G	Directed Evolution of the Taq	A	No	High	No	Yes	1×	Up to 5 kb	0.75–1	Kapa Biosystems/Roche	~1.7 × 10^−5^	- Routine PCR - Fast PCR - Genotyping
Phire Hot-Start II	Archaea of the genus *Pyrococcus*	B	No	High	Yes	No	2×	Up to 20 kb	>2	Thermo Fisher	~1 × 10^−5^	- Hot-Start PCR - Genotyping - High-throughput PCR
Phusion Plus	*Pyrococcus furiosus*	B	No	High	Yes	No	>50×	Up to 20 kb	>3	Thermo Fisher	~4 × 10^−7^	- High-fidelity PCR - Cloning and mutagenesis - Sequencing - Long-range PCR
KAPA HiFi	Directed evolution *Pyrococcus*	B	No	High	Yes	No	>100×	Up to 15–20 kb	>2	Kapa Biosystems/Roche	~2.8 × 10^−7^	- NGS library amplification - Site-directed mutagenesis - Sanger sequencing - Cloning for protein expression or genomic characterization
KOD One	*Thermococcus kodakarensis*	B	No	Ultra-high (1 s/kb for ≤1 kb; 5 s/kb for 1–10 kb)	Yes	No	~80×	Up to 40 kb	>3	Toyobo Co.	~3.0 × 10^−7^	- Direct PCR - Colony PCR - NGS libraries - Site-directed gene mutation
Klentaq1	*Thermus aquaticus*	A	No	Average	No	No	1–2×	Up to 5 kb	1–0.7	DNA Polymerase Technology	~2 × 10^−5^	Amplification from whole blood and serum samples
FIREPol	*Thermus aquaticus*	A	No	Average	No	Yes	1×	Up to 3–5 kb	1.5	Solis BioDyne	2 × 10^−5^	- Routine applications - TA-cloning
ExtremeTaq	*Thermus aquaticus*	A	No	Ultra-high (1–5 s/kb)	No	Yes	1×	Up to 10 kb	0.75–1	Azura Genomics	2 × 10^−5^	- Routine PCR - Colony PCR - TA Cloning - Genotyping - Amplification of GC- or AT-rich regions

## Data Availability

All datasets used and/or analyzed are available from the corresponding author on reasonable request.

## Abbreviations

The following abbreviations are used in this manuscript:

BER	Base excision repair
CFPS	Cell-free protein synthesis
dPCR	Digital PCR
His-tags	Polyhistidine tags
IMAC	Immobilized metal affinity chromatography
NGS	Next-generation sequencing
PCR	Polymerase chain reaction
qPCR	Quantitative PCR
XNA	Artificial nucleic acids
LAMP	Loop-mediated isothermal amplification
LIMA	Linear target isothermal multiple amplification
RCA	Rolling circle amplification
WGA	Whole-genome amplification
CNV	Copy number variation
MDA	Multiple displacement amplification
PLM	Protein language models
FSFP	Few-Shot Learning for Protein Fitness Prediction
